# Prevalence and Characterization of Fluoroquinolone Resistant *Salmonella* Isolated From an Integrated Broiler Chicken Supply Chain

**DOI:** 10.3389/fmicb.2019.01865

**Published:** 2019-08-13

**Authors:** Mingquan Cui, Peng Zhang, Jiyun Li, Chengtao Sun, Li Song, Chunping Zhang, Qi Zhao, Congming Wu

**Affiliations:** ^1^China Institute of Veterinary Drug Control, Beijing, China; ^2^Beijing Advanced Innovation Center for Food Nutrition and Human Health, College of Veterinary Medicine, China Agricultural University, Beijing, China; ^3^Key Laboratory of Detection for Veterinary Drug Residue and Illegal Additive, MOA, College of Veterinary Medicine, China Agricultural University, Beijing, China

**Keywords:** *Salmonella*, fluoroquinolone resistance, *qnrS1*, hybrid plasmid, broiler chicken supply chain

## Abstract

The objectives of this study were to investigate the prevalence and fluoroquinolone resistant *Salmonella* isolated from an integrated broiler chicken supply chain and their molecular characterization. In total, 73 *Salmonella* isolates were recovered from a broiler chicken supply chain in Shanghai. *Salmonella* isolates were tested for susceptibility to 11 antimicrobial agents using the broth dilution method and were characterized using pulsed-field gel electrophoresis (PFGE). Then, the *Salmonella* isolates were examined for mutations in quinolone resistance-determining region (QRDR) of *gyrA*, *gyrB*, *parC*, and *parE*, and were screened for plasmid-mediated quinolone resistance (PMQR) genes. Lastly, we sequenced the plasmids carrying *qnrS1* in six *Salmonella* isolates from three sources (two isolated per source). Among 73 *Salmonella* isolates, 45 isolates were identified as *S*. Indiana, 24 were *S*. Schwarzengrund, 2 were *S*. Enteritidis, and 2 were *S*. Stanleyville. In addition, high rates of resistance were detected for nalidixic acid (41.1%) and ciprofloxacin (37.0%), while resistance to other test agents was diverse (2.0–100%). *S*. Indiana and *S*. Schwarzengrund isolates from different sources exhibited the same PFGE pattern, suggesting that the *Salmonella* isolates possessed high potential to spread along the broiler chicken supply chain. *gyrA* and *parC* exhibited frequent missense mutations. Moreover, *qnrS1* was the most prevalent PMQR gene in the 73 *Salmonella* isolates, and it was found about a new hybrid plasmid. This study concludes a high prevalence of fluoroquinolone resistant *Salmonella* in chicken supply chain, threatening the treatment of *Salmonella* foodborne diseases. In particular, the emergence of a new hybrid plasmid carrying *qnrS1* indicates that the recombination of plasmid carrying resistance gene might be a potential risk factor for the prevention and control strategies of drug resistance.

## Introduction

Salmonellosis, caused by *Salmonella*, is one of the most frequently reported foodborne illnesses worldwide. *Salmonella* is divided into more than 2500 serovars by the White–Kauffman and Le Minor scheme. This classification scheme defines the serogroup according to expression of somatic lipopolysaccharide O antigens, and the serovar by the expression of flagellar H antigens. *Salmonella*, as an important human pathogen, is a potential public health risk.

It has been estimated that *Salmonella* causes about 1.2 million illnesses in the United States every year. Food is a major source of these infections, accounting for 1 million illnesses, 19,336 hospitalizations, and 378 deaths (Scallan et al., 2011). The majority of human infections caused by *Salmonella* is associated with the consumption of food products. Chicken, as one of the most widely consumed meats, is an important reservoir of *Salmonella* (Adu-Gyamfi et al., 2012; Truong Ha and Yamaguchi, 2012). Importantly, antibiotic-resistant bacteria of animal origin could be transmitted to humans (Piddock, 2002; Khemtong and Chuanchuen, 2008), which adversely affect the treatment of salmonellosis. Therefore, it is necessary to monitor the epidemiology and genetic characteristics of *Salmonella* in the food chain.

Fluoroquinolones are widely used to treat salmonellosis in human and animal (Folster et al., 2015). Currently, the main mechanism underlying quinolone resistance is the accumulation of mutations in quinolone resistance-determining region (QRDR) of *gyrA*, *gyrB*, *parC*, and *parE* and plasmid-mediated quinolone resistance (PMQR), which includes five major groups of *qnr* determinants (*qnrA*, *qnrB*, *qnrC*, *qnrD*, and *qnrS*), *aac(6*′*)-Ib-cr* and quinolone extrusion such as *qepA* and *oqxAB* (Strahilevitz et al., 2009). Some studies focused on fluoroquinolone resistance-related determinants about PMQR and QRDR in *Salmonella* derived from humans and animals (Wasyl et al., 2014; Wong et al., 2014). However, comprehensive data regarding fluoroquinolone resistance determinants in *Salmonella* from chicken supply chain are lacking, despite the implication for human health.

Thus, the aims of this study were to investigate the prevalence of *Salmonella* and their molecular characteristics related to fluoroquinolone resistance determinants, including PMQR and QRDR, in the broiler chicken supply chain in Shanghai. These data provide insight into the quantitative risk of resistant *Salmonella* from chicken supply chain.

## Materials and Methods

### Statement of Ethics

This study was carried out in accordance with the ethical guide lines of the College of Veterinary Medicine, China Agricultural University, Beijing. Moreover, before the initiation of this study, formal approval was obtained by the departmental committee of institute. Sampling was carried according to the standard protocols and with prior consent of the farmer/manager of the facilities.

### *Salmonella* Strains and Antimicrobial Susceptibility Testing

*Salmonella* isolates were recovered from three sources including adult broilers, broiler carcasses and retail chicken, representing vertically integrated commercial broiler chicken supply chain in Shanghai City, China. One sample was collected from each animal or meat product as appropriate. Caecal samples from adult broilers were randomly collected at the abattoir. Whole carcasses or meat samples were aseptically obtained from chicken processing chain. Carcasses from the retail chicken source were sampled from the markets. All samples were immediately transported to the laboratory in an insulated ice boxes containing ice packs. Microbiological procedures were performed immediately upon arrival at the laboratory. All test strains were isolated in CHROMagar *Salmonella* agar (CHROMagar Company, Paris, France). Suspected *Salmonella* colonies were confirmed by a PCR assay targeting the *invA* gene (Rahn et al., 1992). *Salmonella* serotyping was conducted by performing the slide agglutination test, using *Salmonella* antisera (S & A Reagents Lab Ltd., Bangkok, Thailand) according to manufacturer’s instructions.

*Salmonella* isolates were subjected to antimicrobial susceptibility tests using standard broth dilution method of minimum inhibitory concentrations according to the guideline of the Clinical and Laboratory Standards Institute [CLSI] (2015a). Antimicrobial agents included 11 antimicrobials (i.e., amoxicillin/clavulanic acid, nalidixic acid, ampicillin, cefazolin, doxycycline, gentamicin, trimethoprim/sulfamethoxazole, chloramphenicol, ciprofloxacin, meropenem, and ceftriaxone). *Escherichia coli* ATCC 25922 was used as a quality control strain. The interpretive category for each isolate (susceptible, intermediate, or resistant) was determined according to the CLSI recommendations (Clinical and Laboratory Standards Institute [CLSI], 2015b).

### Identification of Fluoroquinolone Resistance-Related Determinant

The DNA templates of isolates were prepared using TIANamp Bacteria DNA Reagent Kit (Tiangen, Beijing, China). The extracted DNAs were amplified by PCR assay. The mutations in *gyrA*, *gyrB*, *parC*, and *parE* genes were analyzed as described previously (Eaves et al., 2004). *Salmonella* isolates were screened for *oqxA*, *oqxB*, *qnrA*, *qnrB*, *qnrC*, *qnrD*, *qnrS*, *aac(6*′*)-Ib-cr*, and *qepA* genes. The primers and amplification conditions were described previously (Chen et al., 2012). PCR products were sequenced and identified.

### Pulsed-Field Gel Electrophoresis (PFGE), S1 Nuclease Pulsed-Field Gel Electrophoresis (S1-PFGE), Southern Hybridization, Conjugation and Sequencing by Illumina

All *Salmonella* isolates were analyzed by PFGE method according to a previous protocol for subtyping *Salmonella* (Cui et al., 2016). According to the PFGE profile, six *Salmonella* isolates carrying *qnrS1* were randomly selected from three sources (two isolates per source) for analysis by S1-PFGE and Southern hybridization, as previously described (Zhang et al., 2015). For the conjugation assay, six selected *Salmonella* isolates were used as donor strains, and sodium azide-resistant *E. coli* J53 was used as recipient strains. Both the donor strain and recipient strain were mixed on Luria-Bertani agar at a ratio of 1:3, and 100 μL mixtures were incubated for 16 h at 37°C. Transconjugants were selected on LB supplemented with sodium azide (100 mg/L) and ciprofloxacin (0.5 mg/L). Plasmids DNA were extracted from six *Salmonella* isolates transconjugants using by Wizard^®^ Plus SV Minipreps DNA Purification Systems (Promega, Madison, WI, United States), then been sequenced by Illumina HiSeq 2500 system.

## Results

### Prevalence and Characteristics of *Salmonella* Isolates in the Chicken Supply Chain

**FIGURE 1:**
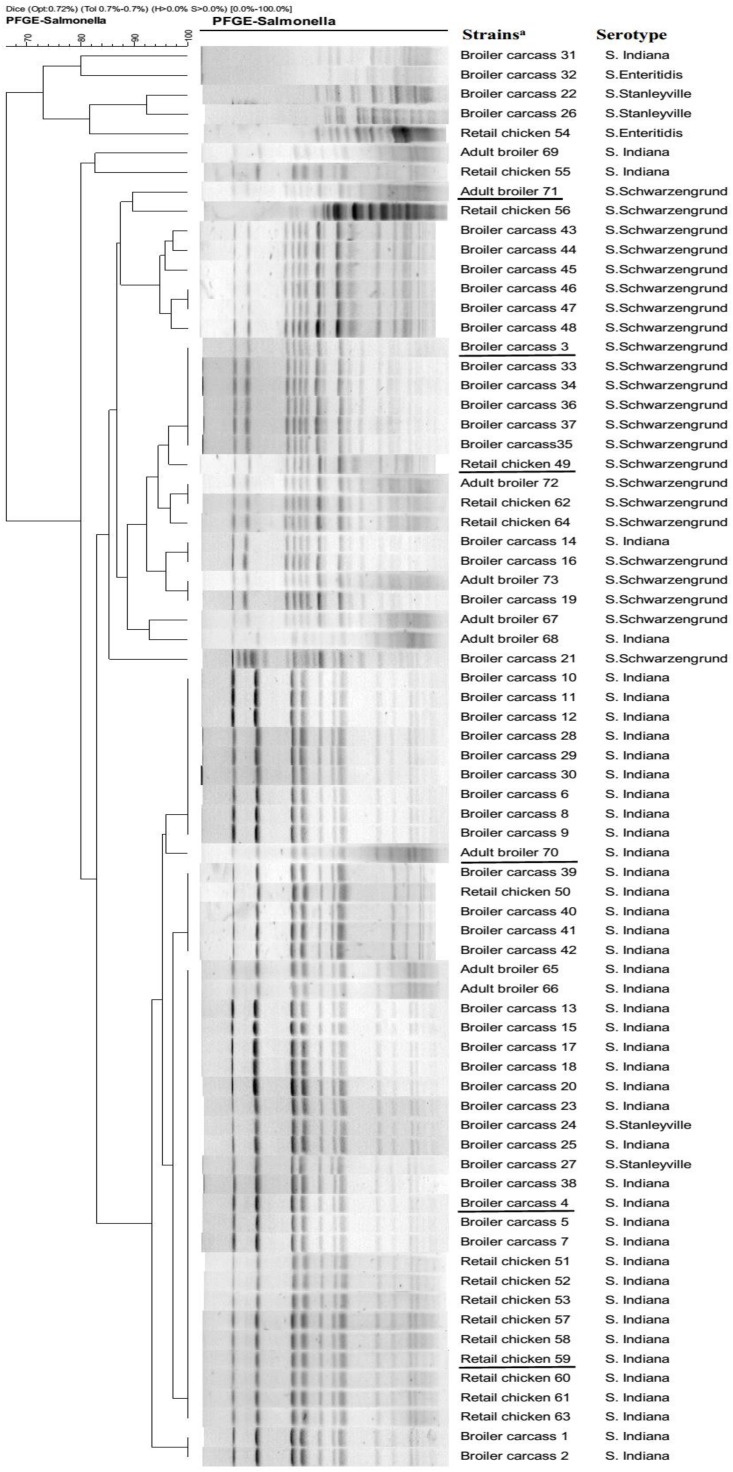
PFGE profiles of *Salmonella* isolates from the broiler chicken supply chain. Strain codes indicate the source of broiler chicken supply chain and the isolate number. ^a^Underlined strains were selected to been sequenced by the Illumina HiSeq 2500 system.

A total of 73 (7.7%) *Salmonella* isolates were recovered from 715 samples. The highest prevalence of (17.5%, 48 isolates) detected in 200 broiler carcass samples, followed by 127 retail chicken samples (8.7%, 16 isolates), while lowest prevalence (2.3%, 9 isolates) of *Salmonella* isolates was observed in 388 adult broiler samples. Among these, 45 isolates were identified as *S.* Indiana, 24 were *S.* Schwarzengrund, 2 were *S.* Enteritidis, and 2 were *S.* Stanleyville. Furthermore, all 73 *Salmonella* isolates were evaluated for susceptibilities to eleven antibiotics. The rates of resistance were 41.1% for nalidixic acid and 37.0% for ciprofloxacin, while, resistance to other test agents varied substantially (amoxicillin/clavulanic acid: 43.8%, ampicillin: 42.5%, cefazolin: 47.9%, doxycycline: 95.9%, gentamicin: 6.8%, trimethoprim/sulfamethoxazole: 100%, chloramphenicol: 43.8%, meropenem: 2.0%, and ceftriaxone: 12.3%). PFGE profiles are shown in Figure 1. Notably, some *S.* Indiana isolates from different sources exhibited the same PFGE pattern. Likewise, some *S.* Schwarzengrund exhibited the same PFGE pattern, suggesting that *Salmonella* isolates have high potential to spread along the broiler chicken supply chain.

### Fluoroquinolone Resistance Determinants in the Chicken Supply Chain

Based on the detail information in Supplementary Table S1, mutations within QRDR of *gyrA*, *gyrB*, *parC*, and *parE* are summarized in terms of serotype in Table 1 The presented data indicated that missense mutations frequently occurred in *gyrA* and *parC*, whereas silent mutations were observed in *gyrA*, *gyrB*, *parC*, and *parE*. Among 73 *Salmonella* isolates from this broiler chicken supply chain, 47 *Salmonella* isolates carried the wild-type (no mutation) within *gyrA* gene, missense mutation (Thr57Ser) within *parC* gene, 15 of which did not exhibit implicated in fluoroquinolone resistance phenotypes. The remaining 26 isolates contained missense mutations within *gyrA* (Ser83Phe and Asp87Asn) and *parC* (Thr57Ser, Ser80Arg). In addition, Table 2 shows the distribution of fluoroquinolone resistance genes (*oqxA*, *oqxB*, *qnrA*, *qnrB*, *qnrC*, *qnrD*, *qnrS*, *aac (6*′*)-Ib-cr*, and *qepA*). Among them, *qnrS1* (30/73) was predominant gene, following by *qnrB1* (22/73), *oqxA* (1/73), *oqxB* (1/73), and *aac* (*6*′*)-Ib-cr* (1/73). The genes sequences of *qnrS1*, *qnrB1*, *oqxA*, *oqxB*, and *aac* (*6*′*)-Ib-cr* were deposited in GenBank. Accession numbers were MK990505, MK990506, MK990507, MK990508, and MK990509, respectively. However, the other fluoroquinolone resistance genes were not observed. In total, 53 of 73 *Salmonella* isolates carried fluoroquinolone resistance genes, and *qnrS1* gene was detected in most *S*. Indiana (23/45), *S*. Schwarzengrund (5/22), and *S*. Enteritidis (2/2) isolates, accounting for the majority *Salmonella* isolates in the broiler supply chain.

**TABLE 1:** Mutations within QRDR of *gyrA*, *gyrB*, *parC*, and *parE* genes in *Salmonella* isolates from broiler chicken supply chain.

		**Serotype**			
		***S*. Indiana**	***S*. Schwarzengrund**	***S.* Enteritidis**	***S*. Stanleyville**
**Gene**	**Mutation type**	**(*n* = 45)**	**(*n* = 24)**	**(*n* = 2)**	**(*n* = 2)**
*gyrA*	Wild type (*n* = 47)	32	13	2	0
	**Ser83Phe**, **Asp87Asn** (*n* = 26)	13	11	0	2
*gyrB*	Lys447, Leu451, Leu462, Ser464 (*n* = 47)	32	13	2	0
	Arg438, Lys439, Leu451, Leu462, Ser464 (*n* = 26)	13	11	0	2
*parC*	**Thr57Ser**, Val67, His75, His77, Asp101, Gly102, Gly104, Ala117, Ser123 (*n* = 47)	32	13	2	0
	**Thr57Ser**, Val67, His75, His77, **Ser80Arg**, Gly104, Ala117, Ser123 (*n* = 26)	13	11	0	2
*parE*	Glu460, His509 (*n* = 27)	14	11	0	2
	Glu460, le464, His509 (*n* = 46)	31	13	2	0

*Missense mutations are marked in bold.*

**TABLE 2:** The distribution of fluoroquinolone resistance genes about PMQR in *Salmonella* isolates from broiler chicken supply chain.

**Genes**	**Serotype**			
	***S*. Indiana**	***S*. Schwarzengrund**	***S*. Enteritidis**	***S*. Stanleyville**
	**(*n* = 45)**	**(*n* = 24)**	**(*n* = 2)**	**(*n* = 2)**
*qnrS1* (*n* = 30)	23	5	2	0
*qnrB1* (*n* = 22)	12	9	0	1
*oqxA* (*n* = 1)	1	0	0	0
*oqxB* (*n* = 1)	1	0	0	0
*aac-Ib-cr* (*n* = 1)	0	1	0	0

**qnrC*, *qnrD*, *qnrA*, and *qepA* were not detected.*

### Novel Plasmid Associated With *qnrS1*

**FIGURE 2:**
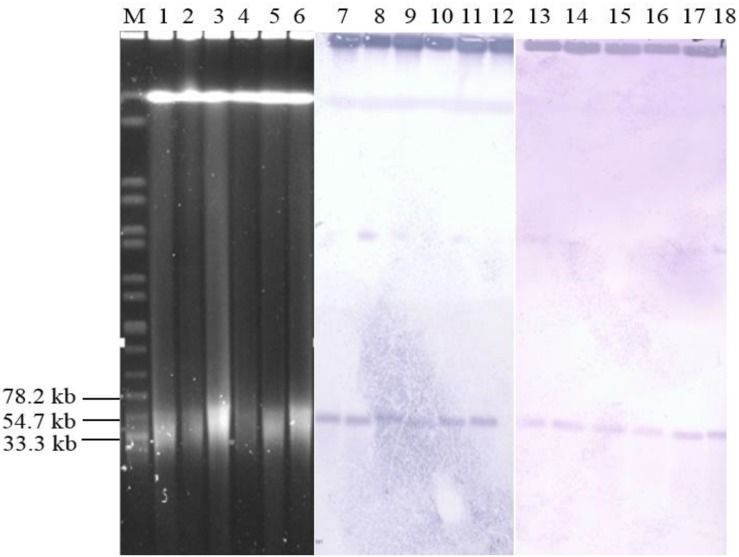
Results of S1-PFGE and Southern blotting. Location of the *qnrS1*-carrying plasmid pSH-01 in *Salmonella* by S1-PFGE (lanes M, 1, 2, 3, 4, 5, and 6) and Southern blot hybridization (lanes M, 7, 8, 9, 10, 11, 12, 13, 14, 15, 16, 17, and 18). Lane M, serotype Braenderup H9812; lane 1 and 7, Adult broiler 70; lane2 and 8, Adult broiler 71; lane 3 and 9, Broiler carcass 3; lane 4 and 10, Broiler carcass 4; lanes 5 and 11, Retail chicken 49; lanes 6 and 12, Retail chicken 59; lanes 13, Adult broiler 70 transconjugant; lane 14, Adult broiler 71 transconjugant; lane 15, Broiler carcass 3 transconjugant; lane 16, Broiler carcass 4 transconjugant; lanes 17, Retail chicken 49 transconjugant; and lanes 18, Retail chicken 5.

The *qnrS1* was predominant gene in the chicken supply chain, thus, six *Salmonella* isolates from three sources (two isolates per source) were randomly selected to analyze the transmission mechanism of *qnrS1* according to the PFGE profile. S1-PFGE and Southern blot analyses (Figure 2) indicated that *qnrS1* is located on a ∼40 kb plasmid, designated pSH-01. Conjugation experiments by filter mating revealed that *qnrS1* could be co-transferred from *Salmonella* isolates to *E. coli* J53. S1-PFGE and Southern hybridization confirmed that the DNA probes specific for *qnrS1* hybridized to the same plasmids with a size ∼40 kb in both *Salmonella* isolates and their transconjugants (Figure 2). Then, the plasmids were extracted from transconjugants and sequenced using the Illumina MiSeq system. The analysis of the sequences showed that the plasmids are extremely similar with more than 99% identity, indicating that the plasmids from the different strains are indeed the same plamids. The NCBI BLAST results showed that the hybrid plasmid carrying *qnrS1* is a new plasmid type (pSH-01, submission number KY486279.1). It was 43,257 bp in length and harbored 51 predicted open reading frames. Furthermore, a plasmid sequence analysis (Carattoli et al., 2014) of pSH-01 indicated that it is an IncR type hybrid plasmid.

**FIGURE 3:**
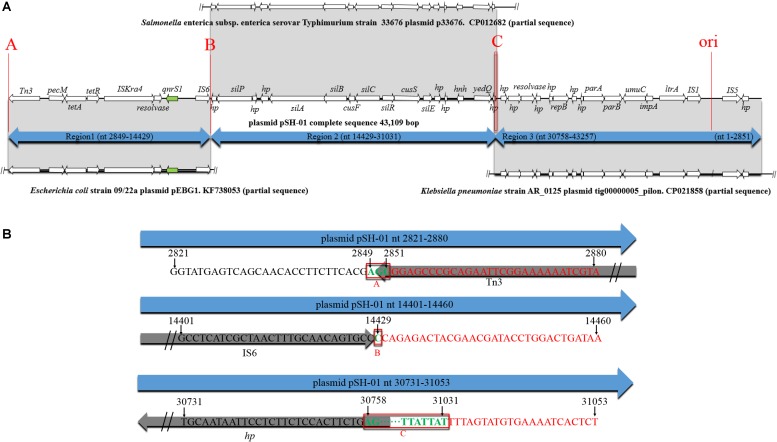
Genetic environment of the *qnrS1* gene in plasmid pSH-01. (A–C) The potential recombination junctions in plasmid pSH-01. **(A)** A structural comparison was made with plasmid pEBG1 from *E. coli* 09/22a, plasmid p33676 from *S.* Typhimurium, and plasmid tig00000005_pilon from *K. pneumoniae* AR_0125. The arrows indicate the positions and directions of transcription of the genes. Regions of 99% nucleotide sequence identity are marked by gray shading. **(B)** Sequence analysis of three recombination junctions (A–C). The bases in black are upstream sequence of recombination junction in plasmid pSH-01. The bases in red are downstream sequence of recombination junction in plasmid pSH-01. The bases in green and in red boxes represent the common region shared by potential hybridized plasmids.

According to the BLAST results of pSH-01 nucleotide sequence against the NCBI database, the two junctions (Figures 3B,C) were often occurred, indicating that the three fragments are usually associated and transferred as a whole. These fragments were almost derived from plasmids. Notably, the genetic features of pSH-01 showed that the fragment carrying *qnrS1* (2849–14429) is derived from plasmids of one *Shigella flexneri* and six *E. coli* isolates (blast query cover: above 99% and blast identity: 99%). Another fragment (nt14429–31031) was derived from plasmids of *Salmonella* and other bacterial isolates, and an additional fragment (30758–43257, 1–2851) was mostly derived from plasmids of *Klebsiella pneumoniae*. The origin of target plasmids covered different bacterial host. According to an alternative sequence analysis, for example, it was postulated that pSH-01 might be hybrids of three plasmids (Figure 3A); the region spanning nt 2849–14429 matched with plasmid pEBG1 (KF738053; nt 37915–26335) of the *E. coli* strain, nt 14429–31031 shared a nucleotide identity of 99% to the plasmid p33676 (CP012682; nt 25123–41725) from *S*. Typhimurium, and nt 30758–43257, 1–2851 shared a nucleotide identity of 99% to the plasmid tig00000005_pilon (CP021858; nt 17585–5015, 4958–1790) from *K*. *pneumoniae* AR_0125. Similar recombination junctions could also be found in another alternative sequence analysis. Therefore, based on sequence analyses of three recombination junctions (Figure 3B), it was postulated that Tn3, IS6 and homologous recombination played important roles in the formation of pSH-01.

## Discussion

In the broiler supply chain in China, there are geographical differences in the dominance of various *Salmonella* serotypes and in the prevalence of fluoroquinolones resistant *Salmonella*. In this study, *S.* Indiana was the most common serotype isolated from the broiler supply chain, which differs from previous results showing that *S*. Enteritidis is dominant in Qingdao and *S*. Weltevreden is dominant in Guangdong, China (Cui et al., 2016; Ren et al., 2016). In addition, several studies have indicated *Salmonella* could be transmitted along the food chain (Nogrady et al., 2008; Hauser et al., 2012). Our PFGE results showed that there is the potential for the transmission of *Salmonella* along the broiler chicken chain. With the emergence of antibiotic-resistant bacteria presenting a serious challenge in human and veterinary medicine globally, there is an abundance of evidence showing that the antimicrobial resistance of *Salmonella* in the chicken supply chain is more possibly attributed to the use of antibiotics in the animal husbandry (Cui et al., 2016). In particular, there are many reports of increasing prevalence of fluoroquinolone-resistant *Salmonella* (Piddock, 2002; Wasyl et al., 2014), which might be a potential risk for human health. In this study, resistance to ciprofloxacin was detected in 37.0% of the *Salmonella* isolates, and this resistance rate was relatively high compared to those of previous reports (Cui et al., 2016; Ren et al., 2016; Nhung et al., 2018).

In this study, the same PFGE pattern was shared among the majority of *Salmonella* isolates (such as *S*. Indiana and *S*. Schwarzengrund isolates), which might suggest they are clones of *S*. Indiana and *S*. Schwarzengrund, respectively. Compared with the QRDR genotypes in non-clones *Salmonella* isolates (Eaves et al., 2004), the genetic diversity of *Salmonella* isolates in this study was lower. In addition, similar to previous investigation (Hopkins et al., 2005), our results indicate that missense mutations occurred frequently in QRDR of *gyrA* and *parC*, which are considered major quinolone resistance determinants in *Salmonella*. In this study, *gyrA* missense mutations (Ser83Phe and Asp87Asn) were detected in 26/73 *Salmonella* isolates and these are considered the major target-site mutations in *Salmonella* (Nüeschinderbinen et al., 2015). Thr57Ser *parC* substitution was frequently observed in the *Salmonella* isolates, and a second substitution (Ser80Arg) in *parC* was also detected in 26 *Salmonella* isolates. Importantly, Thr57Ser *parC* substitution was considered not or doubtfully associated to fluoroquinolone resistance phenotypes (Wasyl et al., 2014). The 15 *Salmonella* isolates with the Thr57Ser *parC* substitution in this study did not show fluoroquinolone resistance phenotypes, in agreement with previous report (Ceyssens et al., 2015). Although mutation types in *gyrA* and *parC* were similar to those in previous studies of *Salmonella*, it is worth noting the high frequency of silent site mutations in QRDR, which might be developed into potential missense mutations (Heisig, 1993; Hopkins et al., 2005). Furthermore, a recent study has shown that mutations in the target genes *gyrA* and *parC* are correlated with an increase of intrinsic fitness in *Salmonella* (Baker et al., 2013). This indicated that the potential risk that *Salmonella* isolates with mutations in *gyrA* and *parC* may naturally maintain during the broiler chicken supply chain, even if fluoroquinolone use was reduced.

In addition, the predominant PMQR gene varies among bacteria from different sources. The most common PMQR gene was *oqxAB* in *E. coli* from chicken (Chen et al., 2012) and in *Salmonella* from retail meat (Lin et al., 2015), *qnrB* in *Enterobacteriaceae* from crows (Halova et al., 2014), and *aac(6*′*)-Ib-cr* in bacteria isolated from sewage and surface water (Osinska et al., 2016). However, this study indicated that *qnrS* was commonly distributed in *Salmonella* isolates from the broiler chicken supply chain, consistent with the high reported rates in *Salmonella* isolated from animals, food, and feed (Wasyl et al., 2014).

The recombination of plasmid, to some extent, can provide a mechanism to improve the diversity of plasmids carrying resistance genes. Recombination of hybrid plasmids frequently occurs at insertion sequence (IS) location (Hudson et al., 2014). Recently, *NDM-5* and *mcr-1* were recombined in the plasmid pCQ02-121 by recombination junctions: IS26 and the *nic* site of *oriT* (Sun et al., 2016). Similarly, in this study, the novel plasmid pSH-01 might arise via recombination junction IS6. The other two recombination junctions involve Tn3 and homologous recombination of sequences. Transposons in the Tn3 family can mediate gene reassortment and genomic plasticity owing to their modular organization, and they contribution substantially to antimicrobial drug resistance dissemination or to endowing environmental catabolic capacities (Nicolas et al., 2015).

Genetic features of pSH-01 showed that only region 2 (nt 14429–31031) could matched the plasmid sequences from clinical *Salmonella* isolates, and the sequences with matches in the other two regions (region 1 and 3) were derived from plasmids from non-*Salmonella* bacteria. The full-length of pSH-01 did not match an individual plasmid in NCBI. Therefore, this is a new plasmid in *Salmonella* isolates from the broiler chicken supply chain, suggesting that the diversity of plasmids carrying the resistance gene might be a potential risk factor for the dissemination of *qnrS1*.

It is worth noting that the new plasmid carrying *qnrS1* presented in the six *Salmonella* isolates (three *S*. Indiana and three *S*. Schwarzengrund) from different sources in the broiler chicken supply chain. This suggests that there was a potential epidemic spread of the plasmid in the *Salmonella* isolates of different serotype from different geographical origin, which is similar to the potential transmission of the plasmids among various serotype of *Salmonella* and diverse geographical location (Li et al., 2016; Wong et al., 2017). Therefore, we should carefully monitor the new plasmid carrying *qnrS1* along the chicken supply chain.

This study provided comprehensive data for the prevalence of *Salmonella* and their fluoroquinolone resistance determinants associated with QRDR and PMQR in the broiler chicken supply chain. Furthermore, we found that *qnrS1*, a transmissible PMQR gene, was prevalent in *Salmonella* isolates from the broiler chicken supply chain. Selective pressure from fluoroquinolones in animals may further promote the recombination and dissemination of the plasmid carrying PMQR genes.

## Author Contributions

CW and MC designed the experiments. MC, PZ, JL, and CS carried out the experiments. MC wrote the manuscript. MC, LS, CZ, and QZ reviewed and revised the manuscript.

## Conflict of Interest Statement

The authors declare that the research was conducted in the absence of any commercial or financial relationships that could be construed as a potential conflict of interest.
